# Supplementary material for: Curcumin Alleviates HMGB1-Mediated Inflammation Through the Signaling Pathway of TLR2-NF-κB in Bovine Ovarian Granulosa Cells
Source: Int J Mol Sci. 2025 Sep 19;26(18):9180. doi: 10.3390/ijms26189180 (PMC12470860; doi:10.3390/ijms26189180)

Fig. 4A β-Actin

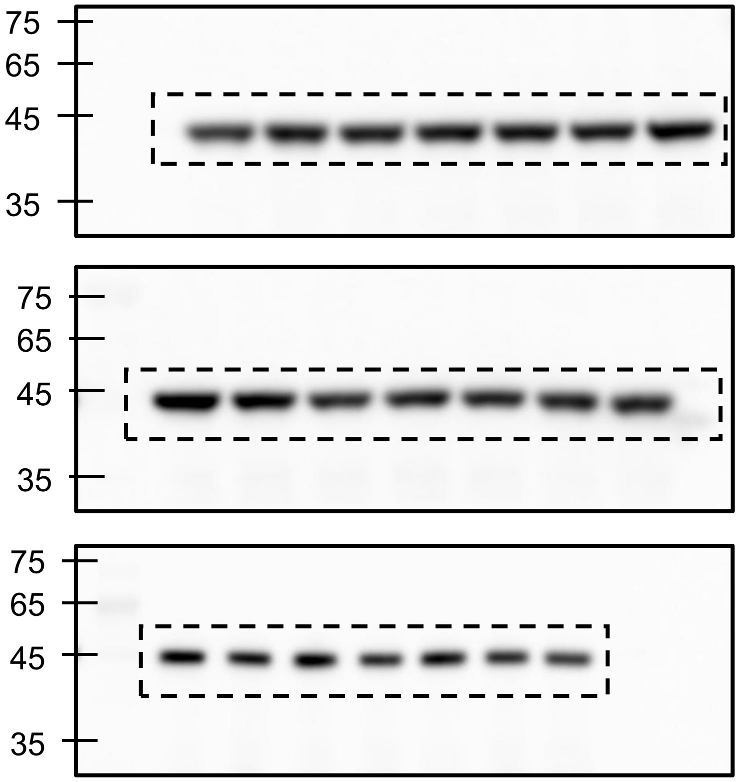

Fig. 4A TLR2

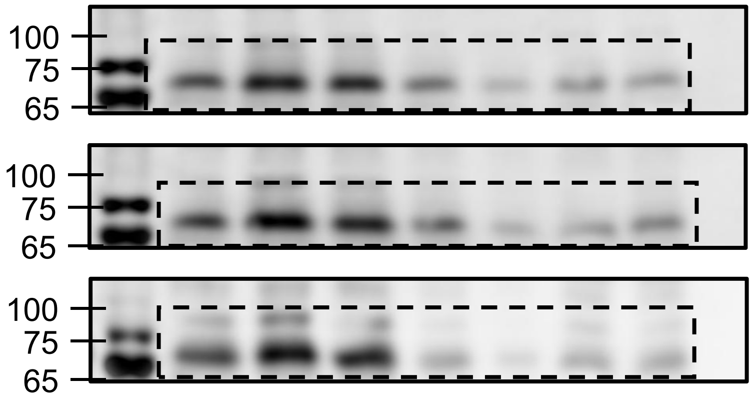

Fig. 4A p-p65

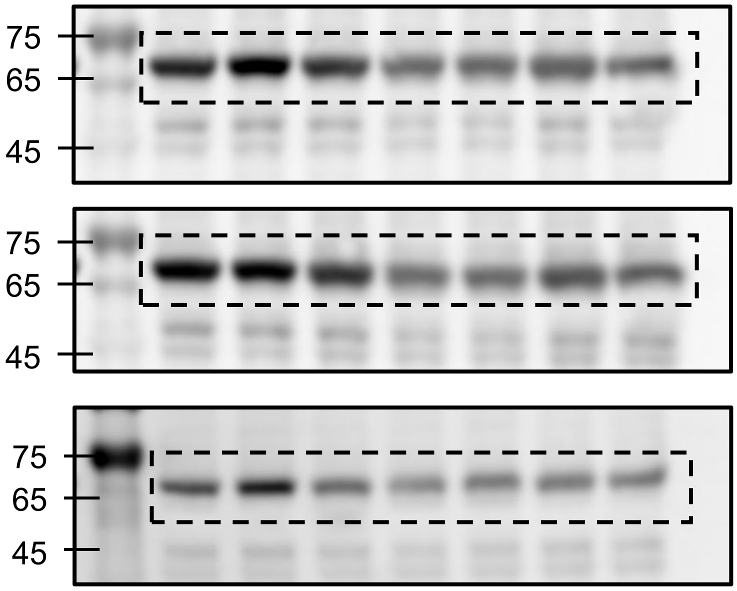

Fig. 4A p65

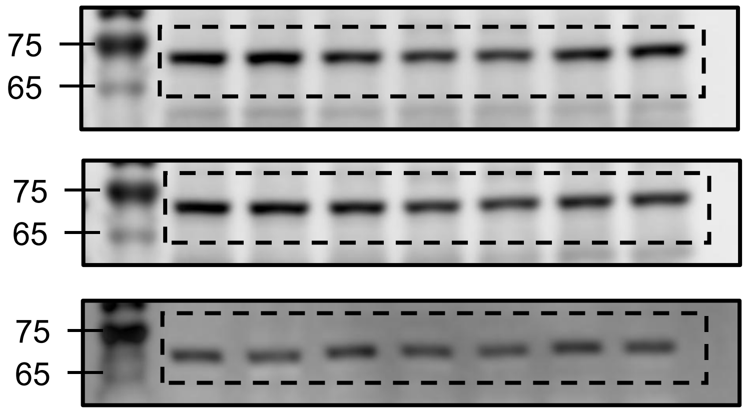

Fig. 4A TLR1

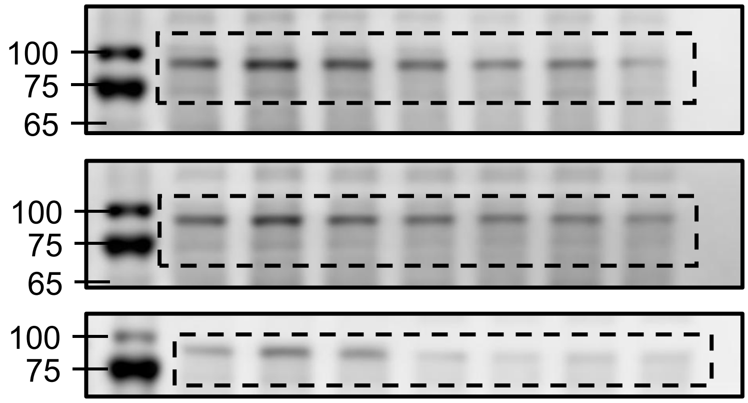

Fig. 4A TLR6

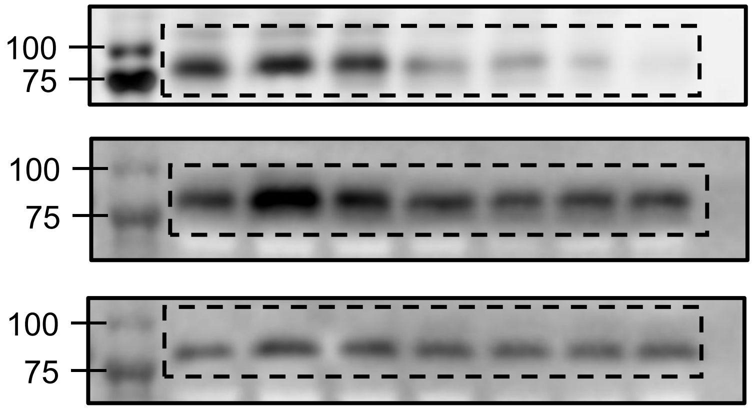

Fig. 6 IP: HMGB1

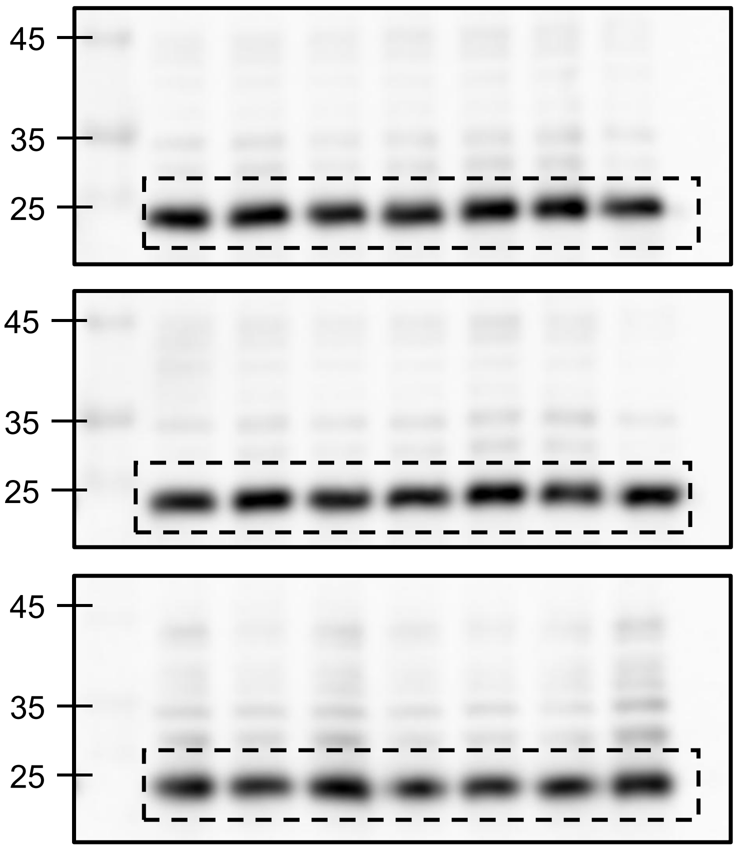

Fig. 6A IP: TLR2

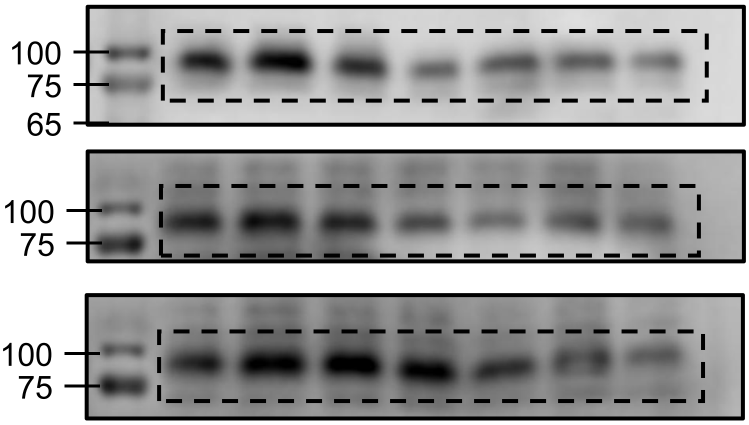

Fig. 6A Input: TLR2

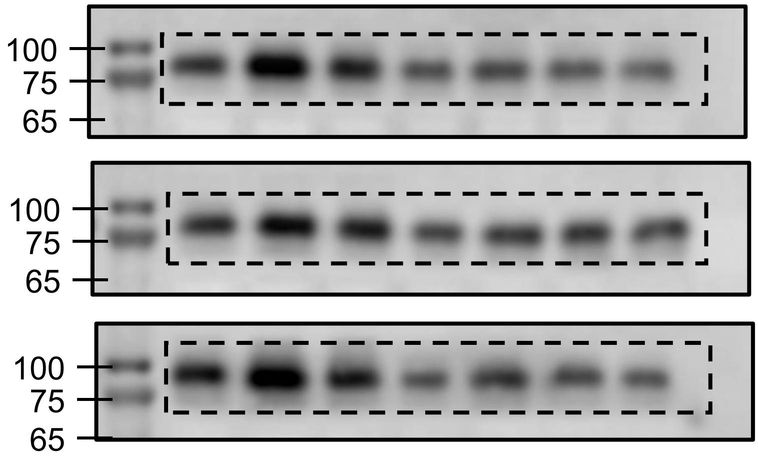

Fig. 6C IP: TLR1

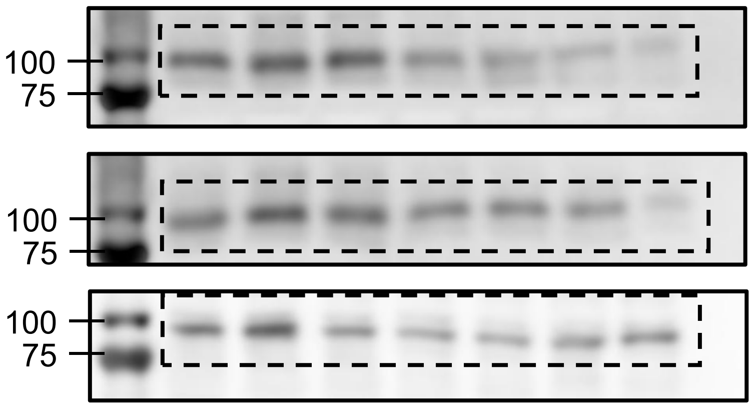

Fig. 6C Input: TLR1

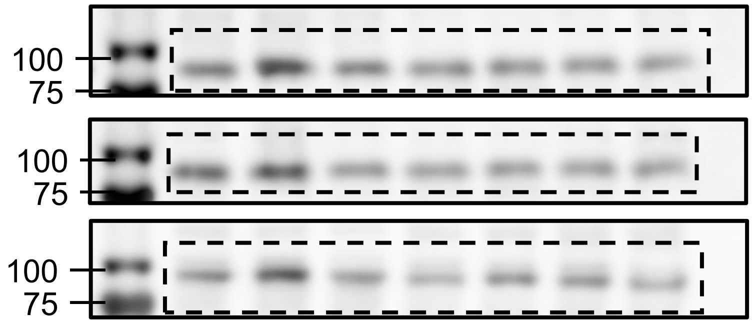

Fig. 6E IP: TLR6

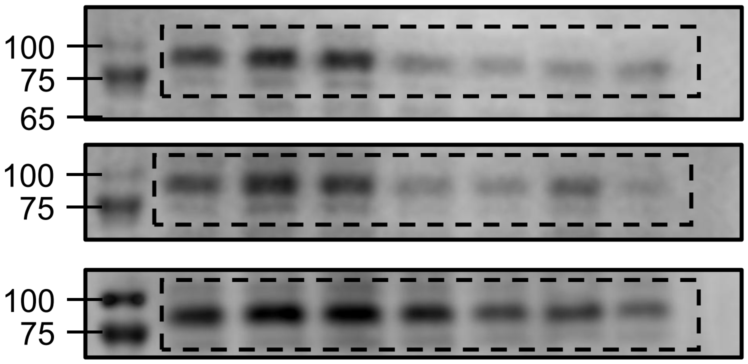

Fig. 6E Input: TLR6

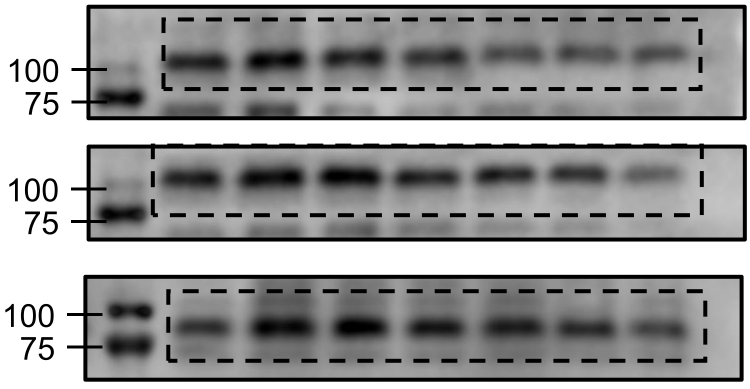

Fig. 6G IP: p-p65

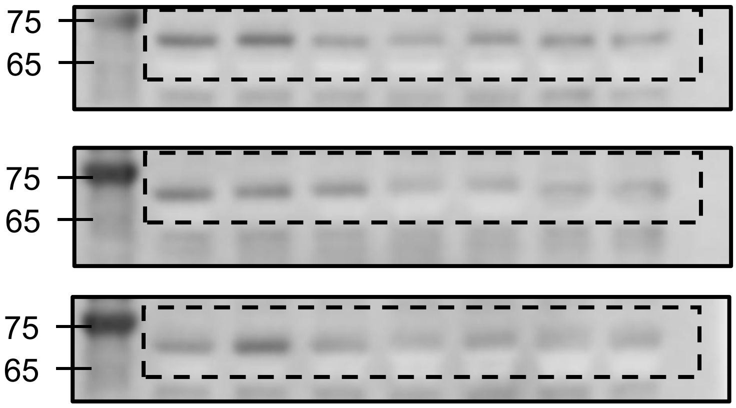

Fig. 6G Input: p-p65

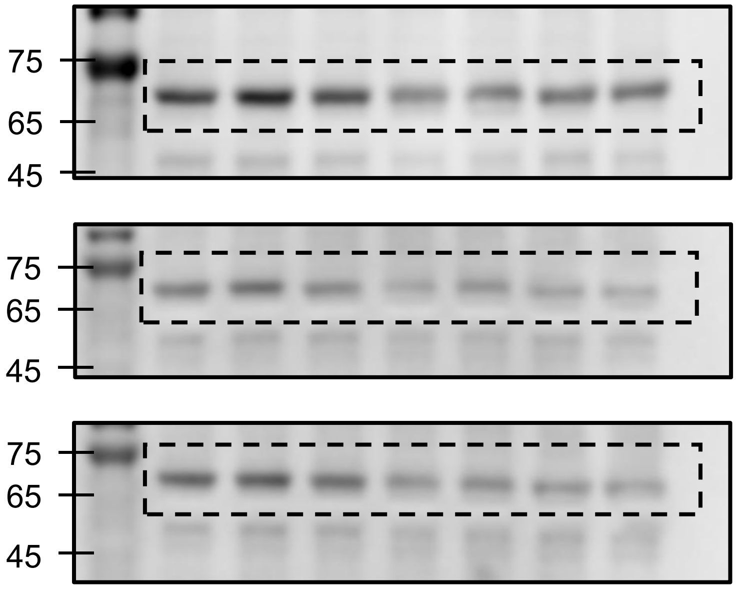

Supplement: Supplementary file 1 [file ijms-26-09180-s001.zip › ijms-3856927-supplementary.pdf]
